# Engineering chimeric antigen receptor-T cells for cancer treatment

**DOI:** 10.1186/s12943-018-0814-0

**Published:** 2018-02-15

**Authors:** Baixin Ye, Creed M. Stary, Xuejun Li, Qingping Gao, Chunsheng Kang, Xiaoxing Xiong

**Affiliations:** 1 Department of Hematology, Renmin Hospital of Wuhan University, Wuhan, Hubei 430060 China; 2 Department of Anesthesiology, Perioperative and Pain Medicine, Stanford University School of Medicine, Stanford, CA 94305-5117 USA; 3 Department of Neurosurgery, Xiangya Hospital, Central south University, Changsha, Hunan 410008 China; 4 Lab of Neuro-Oncology, Tianjin Neurological Institute, Key Laboratory of Post-Neuroinjury Neuro-repair and Regeneration in Central Nervous System, Tianjin, China; 5 Department of Neurosurgery, Renmin Hospital of Wuhan University, Wuhan, Hubei 430060 China

**Keywords:** Tumor ecosystem, Intratumor heterogeneity, T-cell exhaustion, Cancer immunotherapy, Chimeric antigen receptor (CAR) T cell therapy

## Abstract

Intratumor heterogeneity of tumor clones and an immunosuppressive microenvironment in cancer ecosystems contribute to inherent difficulties for tumor treatment. Recently, chimeric antigen receptor (CAR) T-cell therapy has been successfully applied in the treatment of B-cell malignancies, underscoring its great potential in antitumor therapy. However, functional challenges of CAR-T cell therapy, especially in solid tumors, remain. Here, we describe cancer-immunity phenotypes from a clonal-stromal-immune perspective and elucidate mechanisms of T-cell exhaustion that contribute to tumor immune evasion. Then we assess the functional challenges of CAR-T cell therapy, including cell trafficking and infiltration, targeted-recognition and killing of tumor cells, T-cell proliferation and persistence, immunosuppressive microenvironment and self-control regulation. Finally, we delineate tumor precision informatics and advancements in engineered CAR-T cells to counteract inherent challenges of the CAR-T cell therapy, either alone or in combination with traditional therapeutics, and highlight the therapeutic potential of this approach in future tumor precision treatment.

## Background

The diagnostic, monitoring and therapeutic options of cancers remain limited, resulting in a persistent threat to human health, and an urgent need for effective alternative therapeutic measures. Recently, cancer immunotherapies including the anti-programmed cell death protein-1 (PD-1) therapy and the genetically modified T-cell adoptive therapy, have gained headway in the field of cancer therapy [1–6]. In an antitumor immune response, cytotoxic T-lymphocytes (CTLs, a critical subset of effector T-cells) can mediate antitumor immunity through the induction of cytolysis or apoptosis of malignant cells in a human leukocyte antigen (HLA)-dependent manner. However, cancer cells use multiple pathways to evade CTL-mediated antitumor immunity, evolving resistance to currently available combinational therapies, and resulting in cancer relapse or treatment failure [7]. Tumor heterogeneity contributes to the complexity and difficulty of clinical management in antitumor immunotherapy [8–10]. As reported previously, the tumor ecosystem, which is in a state of symbiosis [10], contains heterogeneous cell types including tumor clones with varied spatial, functional as well as genomic characteristics, and associated components that include stromal cells and immune cells [8]. The complex interplay between the multicellular components of the tumor ecosystem play a critical role in tumor development. According to this “tumor ecosystem” theory, therapeutics that target malignant clones, stromal cells and immune cells at multiple layers may represent a potential approach for individualized cancer treatment, highlighting the importance of dissecting the multicellular tumor ecosystem from a *clonal-stromal-immune* perspective [8–11].

Engineered chimeric antigen receptor (CAR) gene-transduced T-cell (CAR-T) therapies have shown great promise in the advancement of individualized clinical cancer immunotherapy. Recently, Novartis’ Kymriah (tisagenlecleucel) became the first FDA-approved CAR-T therapy in the treatment of relapsed or refractory B-cell acute lymphoblastic leukemia in the United State, highlighting the success of CAR-T cell-based immunotherapy [12]. CAR-T cells can be engineered to kill malignant cells specifically or remodel the tumor microenvironment through the release of soluble factors that then regulate the function of stromal cells or immune cells [13–15], providing a powerful tool to target multiple components of the tumor ecosystem. CARs, which contain a fusion protein that is composed of an antibody derived extracellular single-chain variable fragment (scFv) with an antigen recognition moiety and an intracellular T-cell activation domain, can bind to the specific surface tumor antigens and mediate the killing of the tumor cells in an HLA-independent manner. Several clinic trials have demonstrated that CD19-targeted CAR-T-cell-based adoptive immunotherapy leads to a longer remission than current standard combination therapies, particularly in patients with CD19-positive B-cell malignancies including acute lymphoblastic leukemia (ALL), chronic lymphocyte leukemia (CLL) and some lymphomas [14, 15]. In addition to targeting and killing tumor clones directly, CAR-T cells have been utilized as a delivery system to carry effector drugs or proteins to the tumor site locally [16–19]. Despite these advances, functional challenges remain in the effective employment of engineered CAR-T cells for treating malignant diseases, especially for solid tumors. With the recent advancement of Next-Generation sequencing or mass spectrum technologies, treatments targeting tumor ecosystems with high intratumor heterogeneity can adapted to account for tumor clonality and other multicellular components that shape immunosuppressive microenvironment [8, 20, 21]. This potential approach utilizes precision informatics to identify the specific challenges in individual patients, and provides the possibility of precise design and optimization of potential CAR-T cell-based therapeutics or combination therapy in cancer treatment. Genome-editing and molecular engineering technologies also have great potential to equip CAR-T cells with the expression of multifaceted functional genes to counteract these functional challenges [22]. Alone or in combination with other therapeutic modalities, CAR-T cell therapy therefore holds great promise for cancer treatment.

Previously, we have reviewed the mechanisms of tumor immune evasion and the advances in genetically modified T cell-based immunotherapy [23]. In the present review, we will describe the concepts of tumor ecosystem, distinct cancer-immune phenotypes and T-cell exhaustion in immune evasion, providing a deeper and more detailed understanding on tumor immunity from a clonal-stromal-immune perspective. Then, we will review the functional challenges of engineering CAR-T cells, and generalize the framework of engineering and optimizing therapeutic CAR-T cells, alone or in combination with other therapeutics such as chemotherapy, radiotherapy and antibody-based therapy for future cancer treatments.

## Tumor ecosystem and cancer-immune phenotypes

**Fig. 1:**
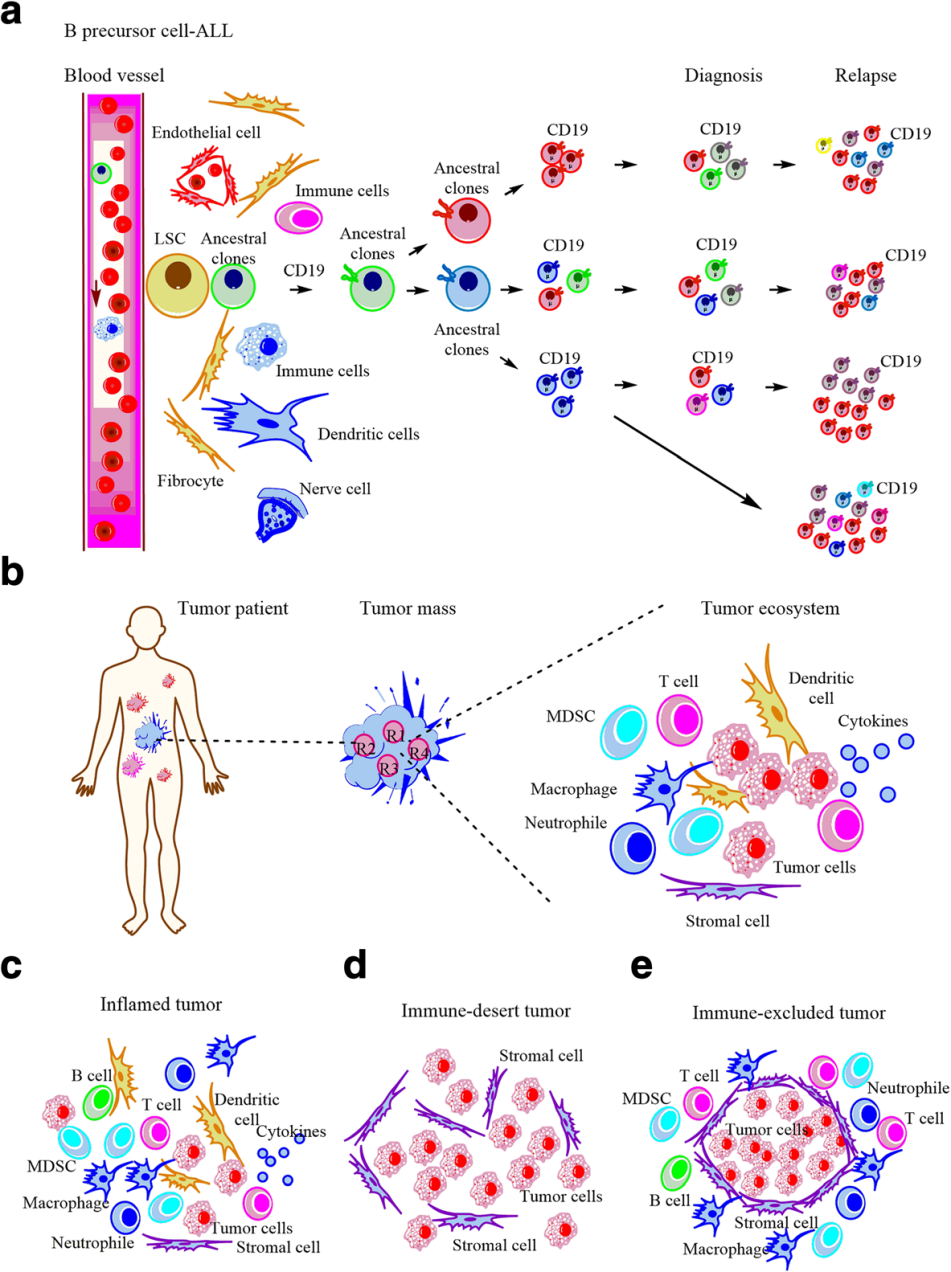
Tumor ecosystem and cancer-immune phenotypes. **a** Oncogenesis of acute lymphoblastic leukemia (ALL) cells with the expression of CD19 in leukemia microenvironment and its clonal evolution from ancestral clones at diagnosis or relapse time point. **b** The tumor ecosystem contains malignant, immune, stromal and endothelial cells in individual tumor patients. MDSC: myeloid-derived suppressor cell. **c-e** Three cancer-immune phenotypes including immune-inflamed (**c**), immune-desert (**d**) and immune-excluded phenotypes (**e**), are related with specific underlying biological mechanisms that may contribute to tumor immune evasion, which then can affect the therapeutic response of immunotherapies

The *tumor ecosystem* is defined by a close interaction and crosstalk between heterogeneous tumor clones and heterogeneous stromal cells (for example, endothelial cells, cancer-associated fibroblasts) as well as immune cells (for example, T- or B- cells, macrophages), which shape tumor development in both the dynamic temporal and spatial dimensions [8]. During the process of tumor generation, tumor initiating clones can interact with their surrounding stromal cells or immune cells within the tumor microenvironment, resulting in the generation of premalignant cells [24]. Upon acquisition of secondary genetic and epigenetic alterations, premalignant clones undergo evolutionary adaptive processes to differentiate into heterogeneous tumor subclones, which are characterized by the expression of different classes of surface markers or intracellular neoantigens. In this complex multicellular ecosystem, all of tumor clones and non-malignant cells exhibit a state of symbiosis, which cooperate to promote tumorogenesis. For example, in B precursor cell-acute lymphocytic leukemia (B-ALL, Fig. 1a), leukemia clones can integrate multiple signals from the niche cells including endothelial cells and immune cells, promoting leukemia evolution, development and relapse [24, 25]. Similarly, in the multicellular ecosystem of solid tumors (Fig. 1b), it was revealed that the branched evolutionary patterns of tumor clones and the heterogeneous suppressive microenvironment, which can be identified at different tumor sites in the individual patients, produce a high intratumor heterogeneity [21, 26–28], challenging the identification of the specific tumor antigens for targeted killing of tumor cells. Moreover, the tumor microenvironment is itself highly heterogeneous in the composition and function of stromal cells or immune cells [8, 20, 21, 29], helping to create an immunosuppressive microenvironment that promotes cancer pathogenesis.

Elucidating the multicellular tumor ecosystem is a first step in employing precision informatics for the design of antitumor immunotherapy. Recently, single-cell sequencing technology has enabled in-depth analysis of the varied types of cells in tumor ecosystem, allowing a more precise determination of heterogeneous tumor clones, their interaction with stromal cells, and the general cancer immune-microenvironment [20, 21]. For example, Zheng, et al. performed deep single-cell RNA sequencing on 5063 single T-cells isolated from hepatocellular carcinoma (HCC) patients, revealing distinctive functional composition of infiltrating T-cells. They found that HCC tissues are distributed with infiltrating regulatory T-cells (T-regs) and exhausted CD8^+^ T cells, the immunosuppressive function of which is closely related with LAYN gene expression in HCC [21]. Dissection of cancer ecosystems through single-cell sequencing technology indicates that tumor infiltrating lymphocytes (TILs) with exhaustion programs exhibit suppressive immune function that kill cancer cells by integrating signals from the tumor microenvironment. Further, Tirosh et al. applied single-cell sequencing technology to profile the malignant, immune, stromal and endothelial cells in tumor tissues for the dissection of multicellular ecosystems of metastatic melanoma, providing a more precise characterization of infiltrating T-cell activation, expansion, exhaustion and variability across patients [8]. These works suggest that a greater understanding of the tumor ecosystem has promoted a shift of cancer therapeutic paradigms from “a clonal perspective” to “a clonal-stromal-immune perspective” [30]. This is represented as a focus not only on tumor clones, but also on the local immunosuppressive microenvironment, towards eliciting an effective antitumor response [11].

Distinct tumors utilize different mechanisms to evade immunity, resulting in the production of distinct cancer-immune phenotypes at a multicellular ecosystem level. In the tumor ecosystem, TILs that traffic and infiltrate into the tumor parenchyma are considered a positive biomarker for cancer treatment and prognosis [31–34]. With the presence of TILs in tumor parenchyma, cancer-immune phenotypes can be described and divided into an immune-inflamed phenotype, an immune-desert phenotype and an immune-excluded phenotype, which is closely related with the therapeutic response to immunotherapies such as anti-PD-1/PD-L1 therapy (Fig. 1c-e) [35, 36]. It has beem previously demonstrated that specific cancer-immune phenotypes have distinct immune response to immunotherapies, suggesting that cancer cells utilize a variety of mechanisms to escape immunity-mediated killing [35]. More specifically, the immune-inflamed phenotype is characterized by the infiltration with many types of immune cells including regulatory T-cells, CD8^+^ or CD4^+^ lymphocytes, suppressor B cells, myeloid-derived suppressor cells and cancer-associated fibroblasts (Fig. 1c), which comprise the tumor ecosystem that precedes the *antitumor* immune response to treatment. Alternatively, the immune-desert phenotype refers to an immune profile described by a paucity of immune cells, especially the cytotoxic T lymphocytes, in either the parenchyma or the stromal of tumor tissue (Fig. 1d). It has been suggested immunological ignorance, tolerance, or a lack of T-cell priming and activation contribute to a lack of pre-existing antitumor response. Finally, the immune-excluded phenotype is characterized by the presence of abundant lymphocytes in the stroma in the absence of infiltration into the parenchyma of tumors. This effect leads to failure of CTL-mediated eradication of tumor clones due to the dislocation of an antitumor immune response, and impaired migration of lymphocytes into the parenchyma of tumor tissue by surrounding stromal cells [35, 37, 38] (Fig. 1e). Either immune-excluded or immune-desert phenotypes are considered non-inflammatory tumors from the clonal-stromal-immune perspective, which can be co-applied as a potential biomarker to predict the immune response upon immunotherapy. For example, it was recently reported that anti-PD-1/PD-L1 therapy can produce a more effective therapeutic response in the immune-inflamed cancers than in non-inflammatory cancers, suggesting that identification of the appropriate cancer-immune phenotype is helpful for the prediction of therapeutic response to immunotherapy [39]. Therefore, a more detailed dissection and description of the tumor ecosystem and deeper understanding of the tumor immune profile is necessary for the development and optimization of novel and effective cancer immunotherapeutics.

## Cancer-immunity cycle and T-cell exhaustion

**Fig. 2:**
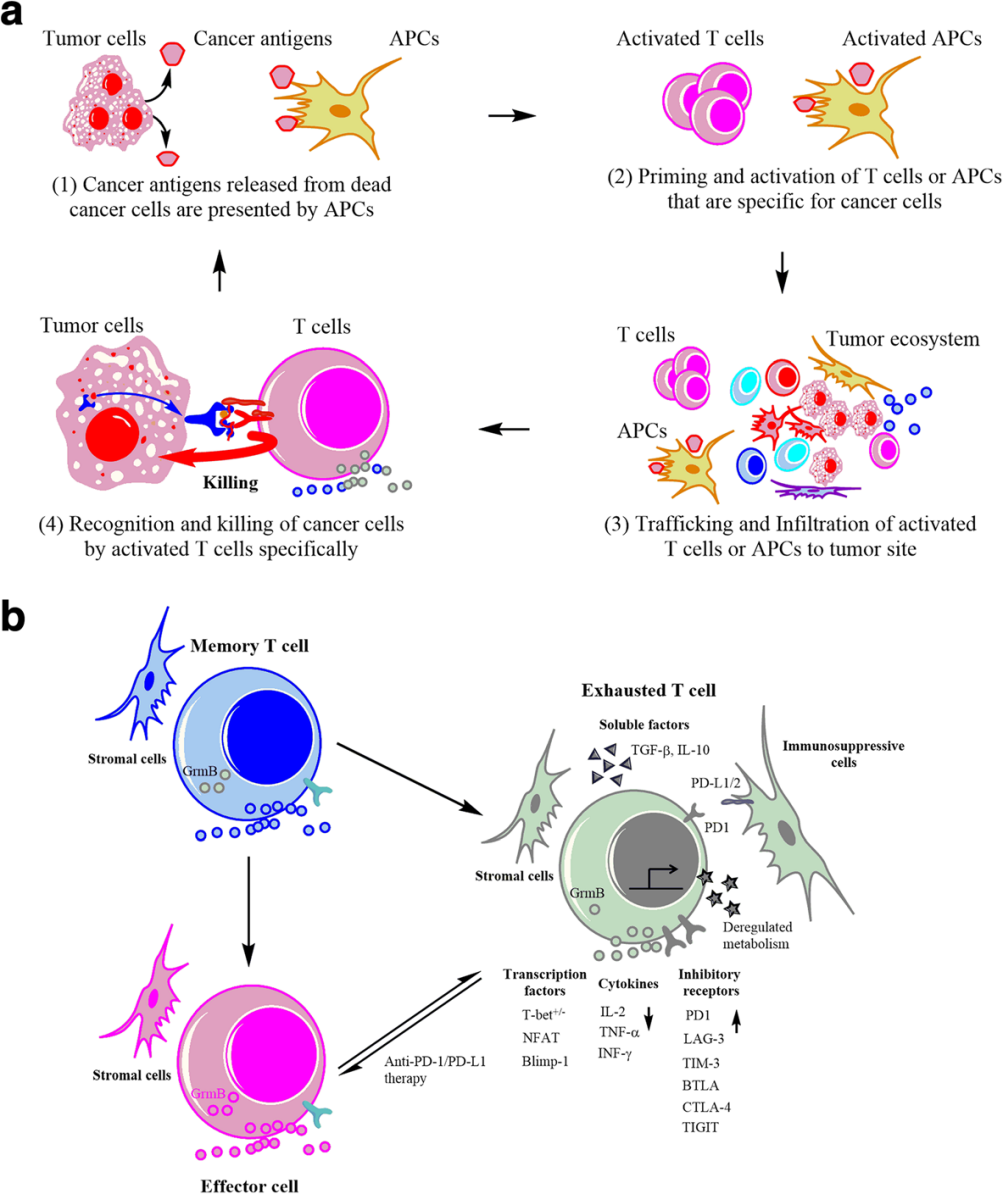
Cancer-immunity cycle and T-cell exhaustion in the tumor ecosystem. **a** Cancer-immunity cycle. Cancer antigens that are derived from destroyed cancer cells are engulfed and presented on the surface of antigen presenting cells (APCs), followed by the priming and activation of T-cells and APCs specific for cancer antigens. Upon activation of effector T-cells or APCs, the effector T-cells bearing cancer antigen-specific TCRs traffic and infiltrate into the tumor sites, recognize cancer cells with the expression of these antigens, and mediate the targeted-killing of cancer cells, which can be processed and presented by APCs in turn. **b** T-cell exhaustion in the tumor ecosystem. T-cell exhaustion refers to the dysfunctional and hypo-responsive state of T-cells, characterized by reduced proliferation and cytokine production, as well as impaired cytotoxicity due to decreased expression of granzyme B. Memory and effector T-cells can differentiate into exhausted T-cells in the tumor ecosystem consisting of tumor clones, stromal cells and immune cells, which can provide internal and external signals for T-cell exhaustion. The internal signals of T-cells in exhaustion are attributed to the enhanced expression of inhibitory receptors (for example, PD-1, CTLA-4, TIM-3, LAG-3, BTLA and TIGIT), the decreased release of immunostimulatory cytokines (IL-2, TNF-α, IFN-γ) and the transactivation of transcription factors (T-bet^−/+^, NFAT, Blimp-1). External signals of exhausted T-cells are from soluble factors (TGF-β, IL-10), stromal cells that can secrete cytokines, immunosuppressive cells (eg. Macrophages and dendritic cells) and deregulated metabolism in the tumor ecosystem. Early exhausted T-cells can be reversed to effector T-cells with treatment of anti-PD-1/PD-L1. TIM-3: T cell immunoglobulin and mucin domain containing-3; BTLA: B and T lymphocyte attenuator; TIGIT: T cell immunoreceptor with Ig and ITIM domains; NFAT: nuclear factor of activated T cell; Blimp-1: B lymphocyte-induced maturation protein 1

In adaptive immunity, APCs including dendritic cells, macrophages and subsets of B cells, process antigens in an HLA-dependent manner, which can be targeted by antigen-specific CTLs providing co-stimulatory signals for priming an antigen-specific CTL response [7, 40]. In the normal cancer-immunity cycle (Fig. 2a), tumor antigens released by destroyed cancer cells can be presented by antigen-presenting cells (APCs), followed by T-cell activation, trafficking and infiltration of effector T-cells into tumor cells, and recognition and targeting of cancer cells with subsequent release of tumor antigens [41]. During the cycle of the T-cell antitumor response, the cancer-immune set point, which is referred to as the threshold that is dependent on the balance between the stimulatory and inhibitory factors in the cancer ecosystem, determines the priming and activation of effective anti-tumor immunity [36]. However, as a result of inhibitory factors in the tumor ecosystem, an effective antitumor T-cell response can be inhibited at several points of the cancer-immunity cycle, thereby promoting tumor cells to evade from immunity-mediated killing. According to the clonal-stromal-immune perspective, the interplay and crosstalk between tumor clones, stromal cells and immune cells in the multicellular ecosystem play a critical role in effector T-cell response and tumor evasion [30]. First, tumor clones with heterogenous mutation-derived neoantigens are involved in the cancer-immunity cycle [7]. Neoantigens that can be processed and presented by APCs can elicit an efficient T-cell response. It was recently reported that the clonal neoantigens exhibit greater efficiency than the heterogenous neoantigens in eliciting an effective immune response [42, 43], suggesting that distinct tumor clones with heterogenous neoantigens can affect the T-cell response. Second, stromal cells, such as endothelial cells and nerve cells, can release tumor progression-related soluble factors, including cyclooxygenase-2 (COX-2) [44, 45], prostaglandin E_2_ (PGE_2_) [45, 46], transforming growth factor-β (TGF-β) [47, 48] and vascular endothelial growth factor (VEGF) [49], which can regulate the T-cell response at multiple stages of cancer-immunity cycle. Third, in the tumor niche, the varied types of immune cells including APCs, T-regs, tumor-associated macrophages (TAMs) and myeloid-derived suppressor cells (MDSCs) [50], constitute the complex immune microenvironment that can shape the cancer-immunity cycle [51]. Therefore, targeting the effector T-cell response in the normal cancer-immunity cycle is critical for the eradication of tumor clones.

However, as reviewed previously, multiple pathways lead to tumor immune evasion and cause failure of treatment [23]. Within the tumor ecosystem, several immunosuppressive factors lead to a poor response of T-cells, termed “T-cell exhaustion,” that mediates tumor immune evasion and serves as an impediment of the normal cancer-immunity cycle [52, 53] (Fig. 2b). The phenotype of T-cell exhaustion is characterized by reduced T-cell proliferation, increased expression of inhibitory receptors (for example, programmed cell death protein-1(PD-1), cytotoxic T-lymphocyte antigen-4 (CTLA-4), lymphocyte activation gene 3(LAG-3), decreased production of stimulatory cytokines (for example, IL-2, IFN-γ, TNF-α.) and compromised cytotoxicity. In general, exhausted T-cells are a distinct lineage from memory or effector CTLs, and can possibly differentiate into defective memory or physical deletion T-cells. From the clonal-stromal-immune perspective, the generation of dysfunctional CTLs in exhaustion can be attributed to multiple complex components of the tumor ecosystem [8, 21], including intracellular signals from inhibitory receptors and altered CTL transcriptional activation, as well as alteration in extracellular signals from tumor, stromal and immune cells such as macrophages, dendritic cells, T-regs and myeloid-derived suppressor cells [52, 53]. Additionally, soluble factors such as TGF-β and IL-10, as well as the metabolic status of the tumor ecosystem such as hypoxia, hypoglycemia and amino acid deletion, can all contribute to T-cell exhaustion [52, 53]. For example, the expression of PD-1 on the surface of CTLs and its ligand PD-L1 on the tumor, stromal or dendritic cells are upregulated, resulting in enhanced PD-1/PD-L1 signals that are considered a critical factor regulating effector function of CTLs to an exhausted phenotype. As reported previously [36], exhausted effector T-cells that express relatively low amounts of PD-1 at early stages can be reversed to a functional state with anti-PD-1/PD-L1 therapy, while the hyper-exhausted effector T-cells with relatively high expression of PD-1 are not recoverable. This observation suggested that exhausted T-cells can be reactivated through early treatment. Therefore, strategies targeting T-cell exhuastion at early stages for reactivation of the normal cancer-immunity cycle are promising for the successful treatment of maligancies [30, 54].

## Advances and challenges in CAR-T cell-based adoptive therapy

### Advances in CAR-T cell therapy

**Fig. 3:**
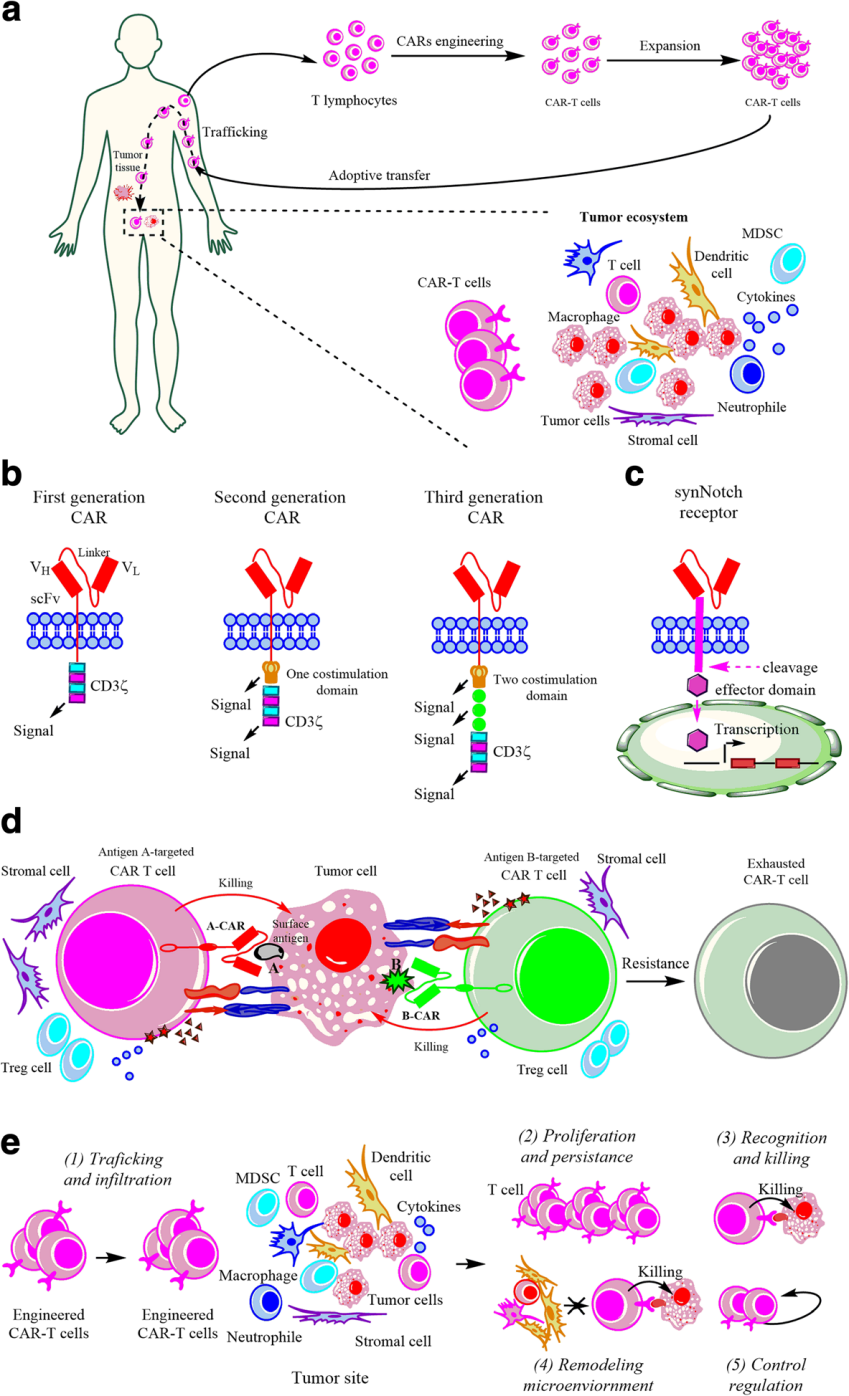
The CAR-T cell therapy and its functional challenges. **a** Procedures of CAR-T cell-based immunotherapy and their trafficking to, and infiltration of, the tumor site. **b** Three generations of CAR structures and the synNotch receptor system. Upon the engagement of tumor surface antigens with extracellular scFv domains, CARs with the intracellular CD3ζ-costimulation (CD28 or 4-1BB) domain can induce native T-cell activation. **c** The synNotch receptor with Notch domain-mediated cleavage and release of specific transcription factors can initiate custom transcription programs upon engagement of surface antigens. **d** CAR-T cells recognize and kill the tumor cells. Different tumor-specific antigen-targeted CAR-T cells can combine to recognize and kill the tumor clones, resulting in enhanced potency and improved clinical outcome. Antigen A-targeted and antigen B-targeted CAR-T cells combine to kill the tumor cells bearing the antigen A and B. Additionally, CAR-T cells can differentiate into the exhausted state, promoting the emergence of therapeutic resistance. **e** Challenges in CAR-T cell-based immunotherapy involve CAR-T cell trafficking and infiltration, adequate CAR-T cell proliferation and persistence, targeted recognition and killing of tumor cells, remodeling of the immunosuppressive microenvironment and self-control regulation

Impaired target-recognition of CTLs to tumor cells can result in immune evasion. Therefore, redirecting the specificity of CTLs to tumor antigens is increasingly recognized as a requirement for cancer immunotherapy [7]. Neoantigen-specific T-cells that naturally exist in cancer patients are difficult to identify and isolate, and the function of T-cells is often disrupted, limiting the application of TIL-based adoptive immunotherapy [22]. However, it was observed that the expression of a novel immunoglobulin-derived zeta chain fusion receptor that is specific for the prostate cancer antigen “prostate-specific membrane antigen (PSMA)” in normal T-lymphocytes confer the specificity and killing-capacity of the T-lymphocytes to prostate cancer cells [55]. This generation of genetically engineered T-lymphocytes transduced with artificial antigen-specific receptors suggests the feasibility of adoptive cell therapy in cancer treatment [54, 56]. Currently, on the basis of technological advancements in genome editing [22, 57], gene transfer and cell culture, the rapid improvement in the generation of genetically modified and patient-derived T-cells bearing CARs has provided a robust platform for the targeted therapy through redirecting the specificity of CTLs against tumor cells [22] (Fig. 3a).

CARs (Fig. 3b), which contain an extracellular ScFv for antibody-like antigen recognition and intracellular signaling domains for T-cell activation from the TCR signaling complex, are genetically modified receptors transduced and expressed in human CTLs for the binding to the surface antigens of tumor cells in their native conformation [14, 15, 58]. In CAR-T cell-mediated immunity, the ScFv can engage surface antigens of tumors directly via antibody-like binding in an HLA-independent manner [15], which is not limited and compromised by impaired processing and presentation of tumor antigens in tumor cells or APCs [14]. The intracellular signaling domains of CAR, which combine the co-stimulatory domains such as CD28 or 4-1BB [59, 60], can be fused with T-cell activating signaling transduction domains such as CD3ζ [14, 54, 61], providing necessary signals for eliciting T-cell activation [15]. As reviewed in our previous report [23], the structure of intracellular signaling domains in CARs has undergone three “generations” of development [15] (Fig. 3b). The 1st-generation CARs contained only CD3ζ transduced into the intracellular signaling domains for transmitting an activator signal to the downstream signaling components. The 2nd-generation CARs contained CD3ζ and one co-stimulatory domain derived from CD28 or 4-1BB, leading to dramatic clinical improvement as a result of release of cytokines and anti-apoptotic factors upon antigen engagement [14, 59]. Further studies indicated that CARs incorporating 4-1BB derived domain, but not CD28, could ameliorate tonic CAR signaling-induced T-cell exhaustion, thus improving CAR-T cell proliferation and persistence [62]. To further improve efficacy, a 3rd-generation CAR construct consisting of multiple co-stimulatory signaling domains including CD28 [59], 4-1BB [60], OX40 [63] and ICOS [64, 65] has been recently developed, although the effect on long-term overall and event-free survival among patient groups remains to be further investigated [14]. Interestingly, W.A. Lim’s group recently demonstrated that a novel synthetic Notch (synNotch) receptor that is engineered in primary T-cells supplies a flexible method to induce customized immune responses [16] (Fig. 3b). Similar with CARs, the synNotch receptor contains a synthetic extracellular recognition domain (e.g., scFv), a core regulatory domain from the cell-to-cell signaling receptor Notch, and a synthetic intracellular domain that can induce downstream transcription. Upon engagement of the extracellular domain of synNotch receptor with a specific antigen on tumor cells, the intracellular domain of the synNotch receptor is cleaved and released into the nucleus of T-cell, thereby activating expression of target genes and inducing an antitumor response. In comparison with CARs that can directly drive T-cell activation, the synNotch receptor functions in a similar antigen-dependent manner but in a T-cell activation-independent manner. As an alternative type of chimeric receptor, the synNotch receptor provides a general platform for engineering primary T-cells to locally deliver non-native therapeutic payloads, remodel the tumor microenvironment and monitor the T-cell immune response. In combination with synNotch receptors, CAR-T cells can be genetically modified to improve their effectiveness and safety profile by sensing environmental factors and modulating T-cell activity [19, 54]. Therefore, CAR-T cells can be engineered not only to mediate specific and effective CTL-mediated killing of tumor cells, but also to exhibit custom response programs in primary T-cells in combination with synNotch receptors [15].

CD19-targeted CAR-T cell-based adoptive immunotherapeutics serve as a successful therapeutic example in patients with relapsed B-cell malignancies, including B precursor cell -ALL, CLL and non-Hodgkin lymphomas in clinic trials [6, 14, 15, 66, 67]. In the development of B-cell malignancies such as B-ALL (Fig. 1a**)** [68], malignant initiating clones with expression of CD19 molecule (ubiquitously expressed in normal B cells) can differentiate into highly heterogeneous subclones at the genetic and epigenetic levels, resulting in difficulties of T-cell therapy for their eradication. For example, given that T-cell recognition of tumor cells occurs in a HLA-dependent manner, malignant subclones become not targetable due to downregulation of HLA molecules, thereby evading T-cell immunity-mediated elimination. Fortunately, nearly all malignant B-ALL clones express CD19 at initial diagnosis or even relapse phase, providing the basis for CD19 as an ideal therapeutic target in CAR-T cell therapy for the following reasons [14]: first, CD19 is expressed in nearly all B-cell malignant clones, which serves as a unique and achievable target for CAR-T cell therapy [14, 69]. Second, normal CD19-expressing B-cells are also possibly killed by CD19-targeted CAR-T cells, but this on-target off-tumor effect does not usually produce clinically unmanageable symptoms [13], providing the basis for clinical applications. Indeed, upon eradication of malignant B-cells, the normal B-cells are also eliminated. Unexpectedly, this loss of normal B-cells is tolerable with combined antibody replacement therapy [14, 54]. Moreover, B-cell aplasia usually occurs in patients with successful CAR-T cell treatment, whereas the persistence of normal B-cells is usually associated with a higher incidence of therapeutic failure [70]. Third, CD19-CAR T-cells showed a delayed early exhaustion, which confers the therapeutic T-cells with the increased capacity of proliferation and persistence [62]. In despite of the early success of CD19-targeted CAR-T cell therapy in B-cell malignancies [14, 54], CAR-related toxicities, including cytokine release syndrome (CRS) and neurological toxicities [23] challenge the development and popularity of CAR-T cell therapy. Additionally, CD19-targeted CAR-T cell therapy also displayed off-target effects and treatment failure due to the disappearance of the targeted epitope on CD19 in about 14% patients, suggesting that antigen loss can be a significant problem in CAR T-cell therapy. Therefore, these observations suggest that current CD19-targeted CAR-T cell approaches require further optimization.

The initial success of CD19-targeted CAR-T cell therapy highlights the importance of choosing optimal surface target antigens in CAR-T cell therapeutics. In addition to CD19 as a CAR target, several other surface tumor antigens have been also considered in CAR-T cell therapy. In ongoing clinic trials, the surface tumor antigens CD20 [71], CD30 [72], CD33 [73], CD123 [74, 75], CD38 [76], CD138 [77], Ig κ light chain and Lewis-Y [78], have been applied as CAR targets, providing a pool of target antigen candidates in cancer treatment, although their clinical outcome remains further determined [15, 78–85]. More recently, cancer-associated Tn glycoform of MUC1, a neoantigen expressed on the cell surface in a variety of cancers, has also been targeted to engineered CAR-T cells and have been shown to be successful in controlling tumor growth in xenograft models of T-cell leukemia and pancreatic cancer [86, 87]. Additionally, CARs have been developed to recognize the intracellular neoantigens that are presented by HLA molecules on the tumor cell surface. For example, a recent report demonstrated that a novel CAR can selectively and specifically target the cell surface complex of the specific liver cancer marker alpha-fetoprotein (AFP) derived AFP158–166 peptide and HLA-A*02:01 [88]. Another novel CAR that exerts a TCR-like function to bind the acute myeloid leukemia (AML)-specific PR1/HLA-A2 complex has been recently developed to efficiently and rapidly kill AML cells in vitro, broadening the application of CAR-T cell therapy especially for those solid tumors with presentation of neoantigens in an HLA-dependent manner [88–94]. Admittedly, in most tumors, intratumor heterogeneity contributes to the variation in the expression and mutation of tumor surface antigens, resulting in antigen loss and tumor relapse. It has also been suggested that CAR-T cells targeting dual or more surface tumor antigens may function to dramatically improve recognition specificity for tumor cells [95, 54, 96] (Fig. 3c). For example, combinational targeting CD19/CD22 antigens can efficiently promote the eradication of the CD19^+^CD22^+^, CD19^−^, and CD22^−^ Pre-B leukemia clones, thus preventing the resistance to single CD19-targeted CAR-T cells in preclinical pre-B cell ALL models [95]. Most recently, Zhou’s group also observed that sequential infusion of CD19 and CD22-targeted CAR-T cells combined to treat the patients with refractory/relapsed B-ALL, resulting in a decreased antigen escape relapse [96]. Therefore, identification and targeting specific tumor antigens show promise to optimize CAR-T cell-based immunotherapeutics.

In addition to directly killing tumor cells, CAR-T cells can deliver antitumor agents to kill tumor clones or remodel the tumor microenvironment in localized tumor sites. As reported by Boice et al. [18], the loss of HVEM (TNFRSF14) receptor gene that serves as a frequently mutated gene in germinal center lymphomas can be disrupted, thus promoting cell-autonomous B-cell proliferation and development of germinal center lymphomas in vivo. To reverse HVEM function, CD19-targeted CAR-T cells were engineered to locally and continuously produce the HVEM ectodomain protein in vivo, and exhibited an enhanced therapeutic activity against xenografted lymphomas. This study illustrated the application of CAR-T cells as a vector to deliver anti-tumor agents to localized and specific tumor sites, implicating the potential for improving efficacy and lowering toxicity of CAR-T cell therapy. This approach may also be potentially combined with synNotch receptor targeting (discussed above) for CD19-targeted or other antigen-targeted CAR-T cells for the delivery of antitumor proteins in localized tumor sites [16, 19]. Such combinatorial potential of CAR-T targeting highlights the clinical potential of their use as both a delivery system and specific antigen-targeting system in cancer treatment.

### Challenges in CAR-T cell therapy

The constantly evolving heterogeneity of cancer cells and their complex microenvironment represent a multifaceted hurdle for cancer immunotherapy and challenges remain in clinical application of CAR-T based approaches. For example, CAR-T cell therapy remains limited in the treatment of solid tumors such as adenocarcinoma and sarcoma [54, 86, 97], and in clinic trials of solid tumors, CAR-T cell therapies have exhibited limited efficacy and severe toxicity [98]. For example, CAR-T cells targeting CD19, folate receptor 1 alpha or CD171 in solid tumors, including lymphoma, ovarian cancer and neuroblastoma, showed severe toxicity, limited efficacy, or induction of host-directed immune responses [14, 15, 58, 99]. These limiting factors in clinic trials underscore the multidimensional challenges of CAR-T cells. Therefore, optimal measures promoting immune enhancement and/or reduced toxicity that utilize genetically modified T cell-based adoptive immunotherapy should be considered.

In general, five major classes of functional challenges should be considered in CAR-T cell therapy [54] (Fig. 3d). First, the trafficking and infiltration of CAR-T cells to tumor sites serves as an important limiting factor for effective CAR-T cell therapy. As described previously, the immunity-desert or immunity-excluded tumors that are characterized by the absence of lymphocytes in the parenchyma of tumors rarely respond to immunotherapeutics such as anti-PD-1/PD-L1 therapy. Similarly, the trafficking and infiltration of effector CAR-T cells determines the therapeutic efficacy in solid tumors, although this might not be a major factor in hematological malignancies. It has been reported that, in solid tumors, the inefficient trafficking and infiltration of CAR-T cells typically results in the failure of treatment [54]. Additionally, CAR-T cells expressing introduced chemokine receptors have been observed in the tumor sites where the chemokines were released [100, 101], suggesting that trafficking and sub-regional infiltration of CAR-T cells is a critical step in their antitumor immune response.

Second, the proliferation and persistence of CAR-T cells influences their clinic efficacy [54]. In antitumor immunity, the expansion of infused CAR-T cells contributes to the required effector-to-target ratio for the targeted killing of tumor cells. However, exhausted CAR-T cells with impaired proliferation and persistence cannot efficiently kill malignant clones, usually leading to treatment failure [62]. 1st-generation CAR-T cells containing only the CD3ζ domain produced limited proliferation and persistence of T-cells [98, 102, 103], while the 2nd-generation CAR-T cells incorporated an additional co-stimulatory signaling domain derived from CD28 or 4-1BB, and promoted T-cell proliferation and persistence [14, 59, 78]. It was previously reported that tonic CAR CD3ζ phosphorylation induced by clustering of CAR scFv in an antigen-independent manner triggers the early exhaustion of CAR-T cells, thus lowering their proliferative and cytokine-producing capacities [62]. Therefore, maintaining the proliferation and persistence of T-cells is also critical for effective CAR-T cell therapy.

Third, targeted-recognition of CAR-T cells is critical for the killing of tumor cells. In antitumor immunity, T-cell recognition and killing of tumor cells in an HLA-dependent manner is critical for an effective immune response, highlighting the importance of targeted-recognition by CTLs. Previously, the generalized view in immunotherapy was that cancer cells can evade the immunity-mediated killing and elimination through the downregulation or loss of HLA molecules expression, resulting in impaired processing and presentation of tumor antigens [7]. To avoid this dilemma, choosing desirable tumor surface antigens is significant for CAR-T cell therapy [15]. However, given that several tumor antigens are co-expressed in normal cells, the ideal specific tumor antigen targets cannot always be easily identified. CAR-T cells targeting non-specific surface antigens can produce cross-reactivity, resulting in the killing of normal cells and severe toxicity [54]. For example, CD19-targeted CAR-T cells can target all of the malignant and normal CD19+ B cells, resulting in B cell aplasia and severe toxicity. Additionally, alternative surface tumor antigens such as CD20, CD30, CD33, CD123, CD38, CD138, Ig κ light chain and Lewis-Y are accompanied with off-target binding. Therefore, identification of desirable surface tumor antigens remains a challenge for CAR-T cell-mediated recognition and killing of tumor cells.

Fourth, according to clonal-stromal-immune perspective, the immunosuppressive microenvironment can limit and impair therapeutic CAR-T cell function. Previous clinical failures in immunotherapy for solid tumors can be partially attributed to the tumor immunosuppressive microenvironment [104, 105]. It has also been observed that the deregulated activity of the enzyme indoleamine 2,3-dioxygenase (IDO) that converted tryptophan into metabolites can shape the immunosuppressive microenvironment and produce an impaired CAR-T cell function [106]. Therefore, the immunosuppressive microenvironment is a important factor limiting the wider applications of CAR-T cell therapy.

Finally, an uncontrolled CAR-T cell response typically leads to severe toxicity and adverse side effects [54, 107]. In clinic trials, the uncontrolled expansion and enhanced activation of therapeutic cells can lead to adverse off-target effects. Historically, control of expansion and function of infused T-cells has not been a primary focus [54]. This dilemma highlights the challenges of designing and engineering the feedback-control regulatory system of T-cells for the optimization of therapeutic timing, strength and location of their activity [107, 108].

## Engineering CAR-T cells for individualized cancer treatment

### Counteracting the functional challenges of CAR-T cells through interventions at multiple dimensions

**Fig. 4:**
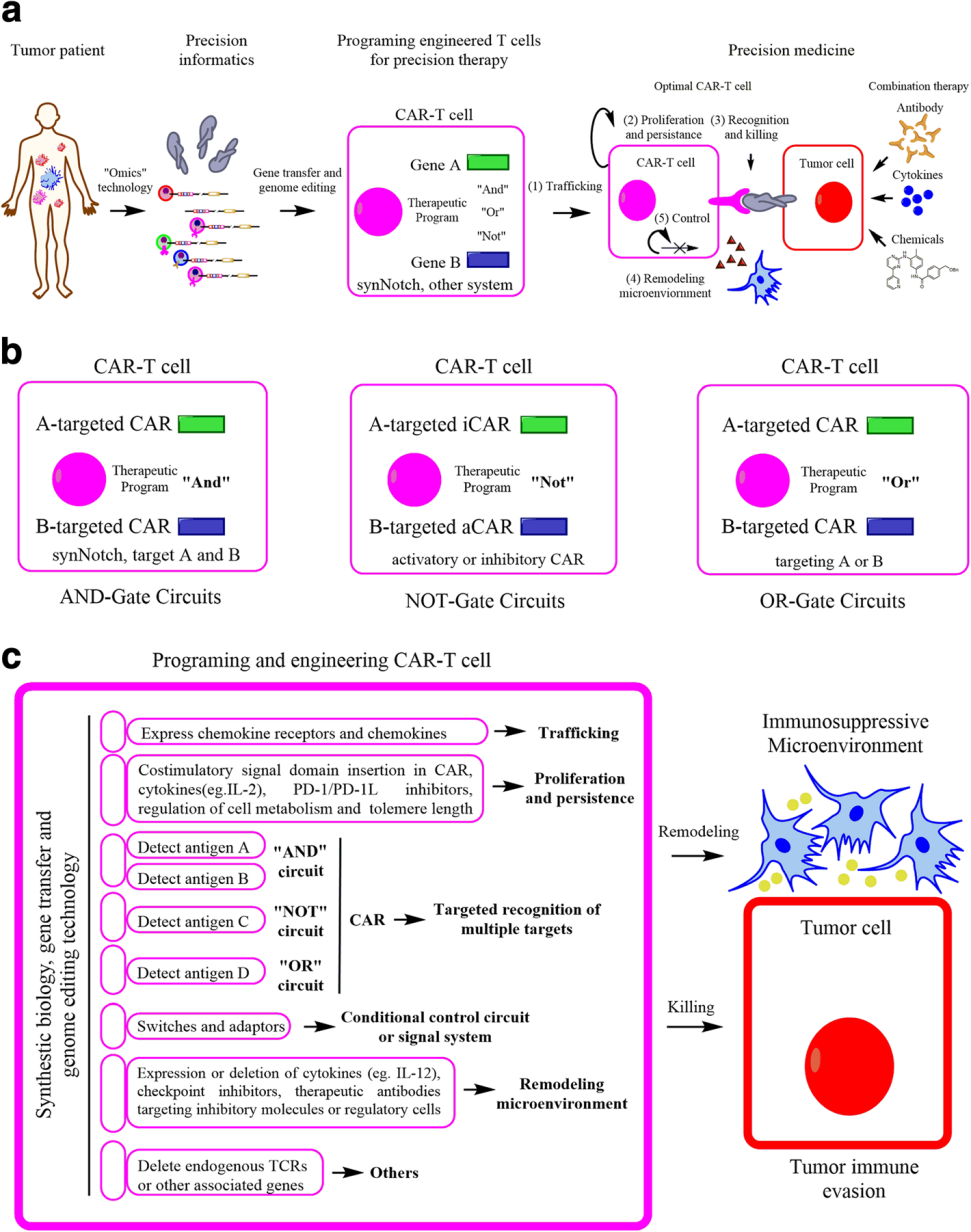
Programming and engineering of CAR-T cell therapy for precision cancer medicine. **a** Procedures of precision informatics and precision therapeutics for precision cancer medicine**.** Through advanced diagnostic technologies including “omics” technologies for more detailed understanding of tumor clones and the microenvironment, precision informatics can counteract the challenges of engineering optimal CAR-T cells, including cell trafficking and infiltration, proliferation and persistence, recognition and killing, remodeling the microenvironment and self-control regulation. As well, a higher order understanding can promote the design of CAR-T cell therapies for use in combination with other therapeutics approaches that target the microenvironment, such as antibodies, chemicals and cytokines. **b** The CAR-based circuits for logical programming include the “AND”, “NOT” and “OR”-Gated circuits, which provide improved recognition specificity through the integration of multiple antigens or combinational antigens. **c** Therapeutic programs for designing and engineering CAR-T cells to improve and optimize their functions include cell trafficking, proliferation and persistence, recognition and killing, remodeling microenvironment and self-control regulation

Basing on our growing understanding of the tumor ecosystem, functional challenges in specific types of cancers can be identified, promoting the advancements in the design and optimization of therapeutic T-cells for precision treatment [54]. More specifically, primary T-cells from tumor patients can be purified and then genetically targeted for counteracting functional challenges. As shown in Fig. 4a, the initial step in CAR-T cell-based precision therapy is to acquire precision informatics of the tumor cells and specific details of their microenvironment. Then, we can more precisely identify optimal therapeutic targets and engineer the therapeutic CAR-T cells with the capacity for targeted recognition and killing. By then applying tumor-specific CAR-T cells expressing multiple functional genes in a localized and controlled manner, we can then build a general platform to counteract the functional challenges in CAR-T cell therapy.

The first consideration is targeted trafficking and infiltration of CAR-T into the parenchyma of tumors, and engineered CAR-T cell can be applied as a tumor-targeting delivery system for the localized and controlled expression of therapeutic factors that can regulate cell trafficking [16, 19]. For cell trafficking and infiltration, previous clinical trials have demonstrated that the incorporation and expression of chemokine receptor genes such as chemokine receptor 4 or 2 (CCR4 or CCR2), in CAR-T cells can promote their trafficking and infiltration into tumor sites, which is then required for effective T-cell mediated killing of tumor cells, especially in solid tumors [100, 109]. Moreover, through recent advances in T-cell chemotaxis and migration, promoting the infiltration of therapeutic CAR-T cells to desirable locations can be potentially achieved by incorporating genes of indicated chemokines or chemokine receptors.

The second important consideration is to improve the proliferation and persistence of CAR-T cells. It has been shown that driving CAR-T cell proliferation and persistence in vivo is a critical limiting factor for durable remission of leukemia, implicating a potential opportunity to improve the function of adoptively transferred T-cells via regulation of T-cell proliferation and persistence [78]. Expression of cytokines such as IL-2 in engineered T-cells, the incorporation of costimulatory signaling domains (eg. CD28 or 4-1BB signaling domains) into CAR molecules [62], prolongation of CAR-T cells’ lifespan through enhancement of telomere length or telomerase activity, modulation of T-cell exhaustion via interruption of PD-1 ligand binding, and reinforcement of lymphocyte metabolism have all been considered for improving T-cell proliferation and persistence [54].

A third critical consideration is improvement in the tumor-targeted recognition ability of CAR-T cells. The great success of genetically modified TCRs or CARs has provided a platform to advance a broader range of intracellular tumor neoantigens. Given that cancer is a heterogeneous disease, bioinformatic analysis suggests that recognizing relatively simple combinations of multiple antigens would dramatically improve the capability of discriminating cancer cells and normal cells. This highlights the importance of CAR-based circuits including AND-Gate circuits, NOT-Gate circuits and OR-Gate circuits, which can integrate information about multiple antigens for the improvement of recognition specificity (Fig. 4b) [54]. In detail, AND-Gate circuits of T-cells bearing two independent antigen-targeting CARs allow the full activation of T-cells upon the engagement to both antigens distributed on the surface of tumor cells, which can improve the specificity of CAR-T cells to tumor clones. Recent advances in 3rd generation CAR-T cells demonstrate a novel AND-Gate recognition mode that is based on the synNotch receptor [16, 17, 19]. In synNotch receptor-mediated circuits, engagement of the synNotch receptor to the corresponding antigen can mediate the intramembrane cleavage to release the intracellular transcriptional activator domain. The application of the synNotch receptor system as an AND-Gate circuit requires binding to the corresponding antigen, which induces the expression of a second receptor (eg. CARs, TCRs or other proteins) that can mediate the killing of tumor cells. In preclinical experiments, synNotch receptor-mediated circuits drove expression of some anti-tumor molecules such as bispecific antibodies and cytokines. In this AND-Gate recognition approach, both antigens on the surface of tumor cells are required for T-cell activation and tumor elimination in a sustained manner, while a single tumor antigen does not. Versus CAR or TCR molecules that mediate the direct killing of tumor cells, synNotch receptor-mediated circuits provide a more precise and localized delivery system to target tumor cells [16, 17, 19]. Alternatively, Not-Gate circuits are based on the combination of an activator CAR (aCAR) mediated by one tumor-killing antigen with an inhibitory CAR (iCAR) by a second normal cell antigen. This approach can be applied in the negative discrimination against normal cell antigens, and effectively reduce cross-reactivity and potential toxicity [110]. Compared with the aCAR bearing an activating signaling domain in their intracellular regions, iCAR intracellular regions are derived from inhibitory receptors such as PD-1 and CTLA-4, and can transduce inhibitory signaling and exert an inhibitory effect on T-cell activation. For example, if a T-cell expressing aCAR and iCAR simultaneously encounters a tumor cell only expressing an aCAR but not iCAR antigen, then that T-cell can kill the select tumor cell. In contrast, if this same T-cell encounters a tumor cell expressing both aCAR and iCAR antigens, then iCAR-mediated inhibitory signaling will override or dampen the aCAR-mediated signaling, preserving that cell [110]. Such CAR-based NOT-Gate circuits therefore provide additional selectivity for therapeutic T-cells in their discrimination of non-cancer signals, resulting in more specific recognition and lower cross-reactivity. In a another iteration, CAR-based OR-Gate circuits are instead based on the CAR molecule bearing two independent antigen recognition domains, which can be activated by one of two different tumor-specific surface antigen ligands and induce the killing of tumor cells [111, 112]. For example, such OR-Gate recognition has been used in the targeting of B-cell malignancies through engineering of CARs specific for CD19 and CD20. Result demonstrated that the CD19 and CD20-targeted CAR-based Gate circuits exhibit equally sensitive and efficient ability of killing tumor cells with or without expression of CD19 [112]. This OR-Gate recognition mode can thereby more efficiently target multiple tumor surface antigens, resulting in prevention of tumor resistance due to loss of tumor antigens. Together, given that the AND-, NOT-, and OR-Gate circuits can be applied to recognize tumor cells specifically through varied modes, it is feasible that co-application with varied CAR-based circuits to target tumor cells can provide even greater precision to cancer targeting in the future.

The fourth major consideration addresses remodeling of the immunosuppressive microenvironment to improve the efficacy of CAR-T cell therapy [18, 29, 113]. According to the tumor ecosystem perspective, the immunosuppressive microenvironment usually found in solid tumors plays a crucial promoting tumor immune evasion and growth [11]. As described previously [11, 114], similar to cancer clones, the immunosuppressive microenvironment is also heterogeneous, suggesting that appropriate and personalized countermeasures to target the specific tumor microenvironment is required for precision approaches. To block the effect of PD-1 mediated signaling, the extracellular domain of PD-1 has been previously fused to intracellular co-stimulatory domains, generating a chimeric receptor that can engage the normally suppressive PD-1 signal, but instead transduces a signal to enhance T-cell activity. Alternatively, removal of the PD-1 receptor in CAR-T cells through CRISPR genome-editing technology has been demonstrated to enhance the T-cell response in the absence of PD-1 ligand-mediated signaling, suggesting that checkpoint inhibition can shape the immunosuppressive microenvironment alternatively via PD-1 or CTLA-4 mediated signaling. Additionally, incorporation of synNotch-based signaling systems, CAR-T cells can locally express and release a variety of payloads such as proinflammatory cytokines (for example, IL-12), checkpoint inhibitors and bispecific antibodies, which can also be used to remodel the tumor immunosuppressive microenvironment [16, 17, 19].

Finally, the fifth consideration is design and implementation of desirable feedback control circuits that restrict the toxicity or hyperactivity of CAR-T cells [107, 115–117]. Toxicity and unregulated expansion and proliferation of CAR-T cell based approaches can lead to catastrophic clinical failure in vivo [118–120]. Technologies that can modulate transgene expression in a controlled manner, as well as activate or terminate T-cell function in vivo, are essential for the generation of CAR-T cells with controlled activity. To take advantage of immune stimulatory factors in boosting anti-cancer T-cell responses, it is also of crucial importance to control the timing and magnitude of the factor expression. For example, the level of IL-6 release is an important indicator for evaluating the toxicity of cytokine release syndrome in CAR-T cell therapy, implying that a feedback circuit for detecting IL-6 levels is necessary for monitoring therapeutic toxicity [54]. To produce a controlled response of therapeutic T-cells, the following approaches have been considered: inducing suicide or elimination switches that can be potentially triggered by physicians to eliminate hyper-reactive T-cells at indicated time points [107, 108, 117], drug-controlled ON-Switch CARs that can control T-cell activity in a specific drug-dependent manner [54, 121], or adaptor-mediated CARs that can target specific tumor cells through the binding of specific adaptor antibodies [122, 123]. In summary, for an effective and successful CAR-T-cell based immunotherapy, it is necessary to identify the critical functional challenges to address the target disease from multiple different dimensions: promoting logical design and engineering of CAR-T cells with desirable characteristics and functions in trafficking, proliferation and persistence, recognition and killing of tumor cells, remodeling the tumor microenvironment and regulatory control (Fig. 4c) [54].

### Genome-editing technology in engineering CAR-T cells

In addition to gene transfer technologies widely used in cell biology, recent advancements in genome-editing technologies has led to enhanced efficacy and reduced toxicity of CAR-T cell therapy [124]. Genome-editing technologies that mainly include zinc-finger nucleases (ZFNs), transcription activator-like (TAL) effector nucleases (TALEN) and clustered regularly interspaced short palindromic repeats (CRISPR) approaches [125–127], provide a feasible method to add or remove functional genes in therapeutic T-cells, thereby engineering CAR-T cells with a variety of characteristics [22, 128]. For example, exogenous TCRs expressed in engineered CAR-T cells can mismatch and compete with their endogenous TCRs, thus producing decreased activity and severe toxicity [129]. Engineered CAR-T cells with disruption of the endogenous TCRα and TCRβ genes by ZNFs have been shown to maintain anti-tumor activity in vivo, but did not produce off-target reactivity [57], demonstrating that genome-editing technology can equip CAR-T cells with an improved biosafety profiles. Recently, CRISPR/Cas9 genome-editing technology has been applied to redirect a CD19-specific CAR to the T-cell receptor α constant (TRAC) locus, resulting in uniform CAR-expressing T-cells [126]. Compared with conventionally generated CAR-T cells, these CRISPR/Cas9-edited CAR-T cells showed an enhanced potency, delayed differentiation and exhaustion of effector T-cells, highlighting the tremendous potential of genome-editing technology in the advancement of CAR-T cell therapy. Therefore, genome-editing technology is a highly promising approach to optimize the CAR-T cell therapy in the future.

### Combination of CAR-T cells with other therapeutics for cancer treatment

To improve efficacy and decrease toxicity, the combination of CAR-T cell therapy with other therapeutics such as immune checkpoint inhibitors, chemotherapy, and other adjunct treatments have demonstrated promise (Fig. 4a) [130]. For example, prior to CAR-T cell therapy, lympho-depletion chemotherapy may promote antitumor activity via enhancing the T-cell response, killing suppressive immune cells, and optimizing the antigen presentation process [14]. Previous studies have also demonstrated that immune checkpoint related antibodies, such as anti-PD-1, PD-L1 and CTLA-4, when combined with CAR-T cells in solid tumors such as melanoma, and lung cancer, may produce an enhanced T-cell activity, with promising clinical outcomes [131].

## Conclusion

CAR-T cell-based immunotherapy represents as a novel, powerful approach for tumor treatment, however several hurdles remain for therapeutic optimization. First, from the clonal-stromal-immune perspective, precision informatics of the tumor ecosystem (provided by “omics” approaches) is required for the successful programing and engineering of CAR-T cells. Armed with this information, we can then seek the answers to the following questions: what are the desirable tumor antigens that can be targeted by CAR-T cells? What are the immunosuppressive factors in the tumor ecosystem? How can we define the tumor’s distinct cancer-immunity phenotype? What mechanisms do CAR-T cells use to evade immunity? This helps to identify the main functional challenges of CAR-T cell therapy so comprehensive measures at multiple dimensions can then be undertaken, including T-cell trafficking and infiltration, proliferation and persistence, targeted recognition of tumor antigens, remodeling the microenvironment and self-control regulation. Finally, in combination with other therapeutic measures, including genome-editing technology, immune checkpoint inhibitors, chemotherapy and cytokine treatment, an optimal, precision, CAR-T cell therapy can be designed and employed for individual cancer therapy. While CAR-T cell-based immunotherapeutics still face many challenges, we are beginning to define the horizon for an individualized cancer treatment.
